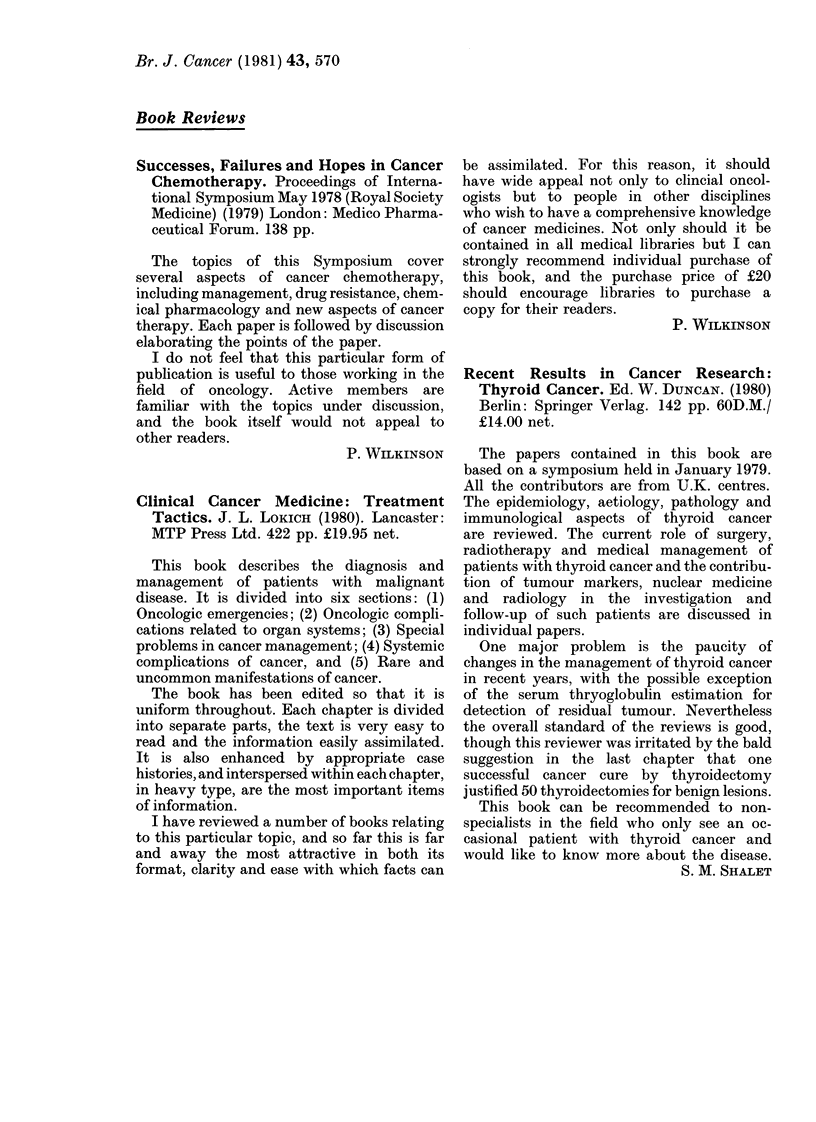# Clinical Cancer Medicine: Treatment Tactics

**Published:** 1981-04

**Authors:** P. Wilkinson


P. WILKINSON

**Clinical Cancer Medicine: Treatment Tactics.** J. L. LOKICH (1980). Lancaster: MTP Press Ltd. 422 pp. £19.95 net.

This book describes the diagnosis and management of patients with malignant disease. It is divided into six sections: (1) Oncologic emergencies; (2) Oncologic complications related to organ systems; (3) Special problems in cancer management; (4) Systemic complications of cancer, and (5) Rare and uncommon manifestations of cancer.

The book has been edited so that it is uniform throughout. Each chapter is divided into separate parts, the text is very easy to read and the information easily assimilated. It is also enhanced by appropriate case histories, and interspersed within each chapter, in heavy type, are the most important items of information.

I have reviewed a number of books relating to this particular topic, and so far this is far and away the most attractive in both its format, clarity and ease with which facts can be assimilated. For this reason, it should have wide appeal not only to clincial oncologists but to people in other disciplines who wish to have a comprehensive knowledge of cancer medicines. Not only should it be contained in all medical libraries but I can strongly recommend individual purchase of this book, and the purchase price of £20 should encourage libraries to purchase a copy for their readers.